# Approaches to Using the Chameleon: Robust, Automated, Fast-Plunge cryoEM Specimen Preparation

**DOI:** 10.3389/fmolb.2022.903148

**Published:** 2022-06-23

**Authors:** Talya S. Levitz, Miriam Weckener, Ivan Fong, James H. Naismith, Catherine L. Drennan, Edward J. Brignole, Daniel K. Clare, Michele C. Darrow

**Affiliations:** ^1^ Department of Biology, Massachusetts Institute of Technology, Cambridge, MA, United States; ^2^ Structural Biology, The Rosalind Franklin Institute, Harwell Science Campus, Didcot, United Kingdom; ^3^ Division of Structural Biology, The Wellcome Centre for Human Genetics, University of Oxford, Oxford, United Kingdom; ^4^ SPT Labtech, Melbourn, United Kingdom; ^5^ Department of Chemistry, Massachusetts Institute of Technology, Cambridge, MA, United States; ^6^ Howard Hughes Medical Institute, Massachusetts Institute of Technology, Cambridge, MA, United States; ^7^ MIT.nano, Massachusetts Institute of Technology, Cambridge, MA, United States; ^8^ Diamond Light Source Ltd., Harwell Science Campus, Didcot, United Kingdom; ^9^ Artificial Intelligence and Informatics, The Rosalind Franklin Institute, Harwell Science Campus, Didcot, United Kingdom

**Keywords:** cryoEM specimen preparation, vitrification, automation, self-wicking grids, air-water interface issues, preferred orientation, denaturation/dissociation

## Abstract

The specimen preparation process is a key determinant in the success of any cryo electron microscopy (cryoEM) structural study and until recently had remained largely unchanged from the initial designs of Jacques Dubochet and others in the 1980s. The process has transformed structural biology, but it is largely manual and can require extensive optimisation for each protein sample. The chameleon instrument with its self-wicking grids and fast-plunge freezing represents a shift towards a robust, automated, and highly controllable future for specimen preparation. However, these new technologies require new workflows and an understanding of their limitations and strengths. As early adopters of the chameleon technology, we report on our experiences and lessons learned through case studies. We use these to make recommendations for the benefit of future users of the chameleon system and the field of cryoEM specimen preparation generally.

## Introduction

**FIGURE 1:**
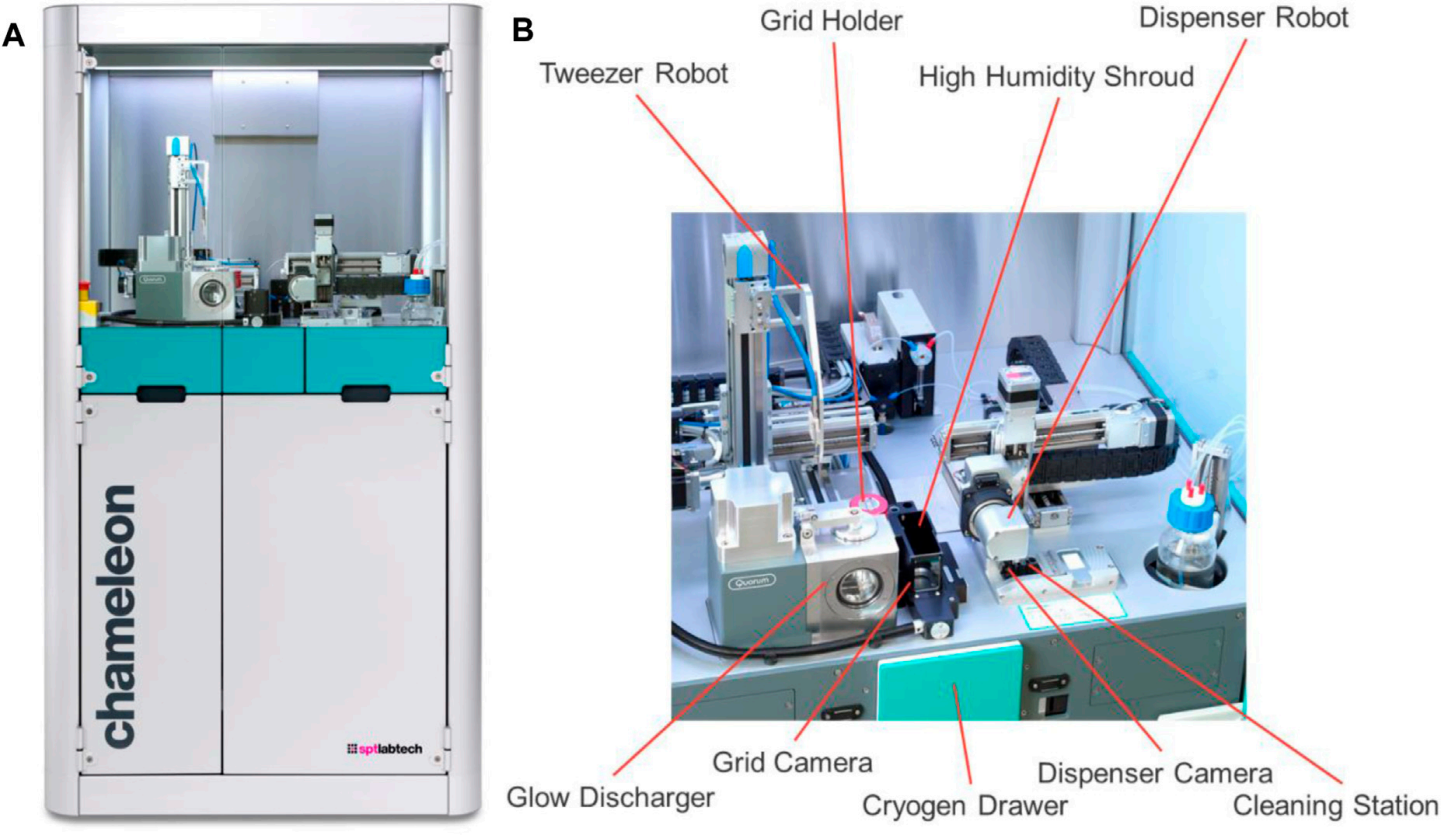
Overview of the chameleon system. **(A)** The entire system with the robotic equipment at the top and the built-in cupboard underneath. **(B)** A close-up of the top deck with the robotic equipment needed for specimen preparation highlighted.

Electron microscopy of biological molecules requires that they are frozen or chemically fixed prior to their placement under high vacuum and exposure to damaging electrons (Taylor and Glaeser, 1976; Henderson, 1990). Crystalline ice strongly diffracts electrons, obscuring the image of the sample, and must be avoided. Thus, the vitrification of biological samples is a key step in electron microscopy. The chameleon is a blot-free, pico-litre dispensing instrument designed to automate the specimen preparation process for cryo electron microscopy (cryoEM) (Darrow et al., 2019) and is based on the original Spotiton design (Wei et al., 2019). It is a free-standing instrument, approximately 1.7 m tall (Figure 1) with an upper cabinet that houses the robotic equipment needed to handle the sample and grids and an on-board glow discharger. The cryogens are contained in a motorised drawer, and supporting systems (humidifier, cleaning, waste, etc.) and storage space are available in the built-in cupboard below. A computer monitor, keyboard and mouse sit to one side of the chameleon system.

**FIGURE 2:**
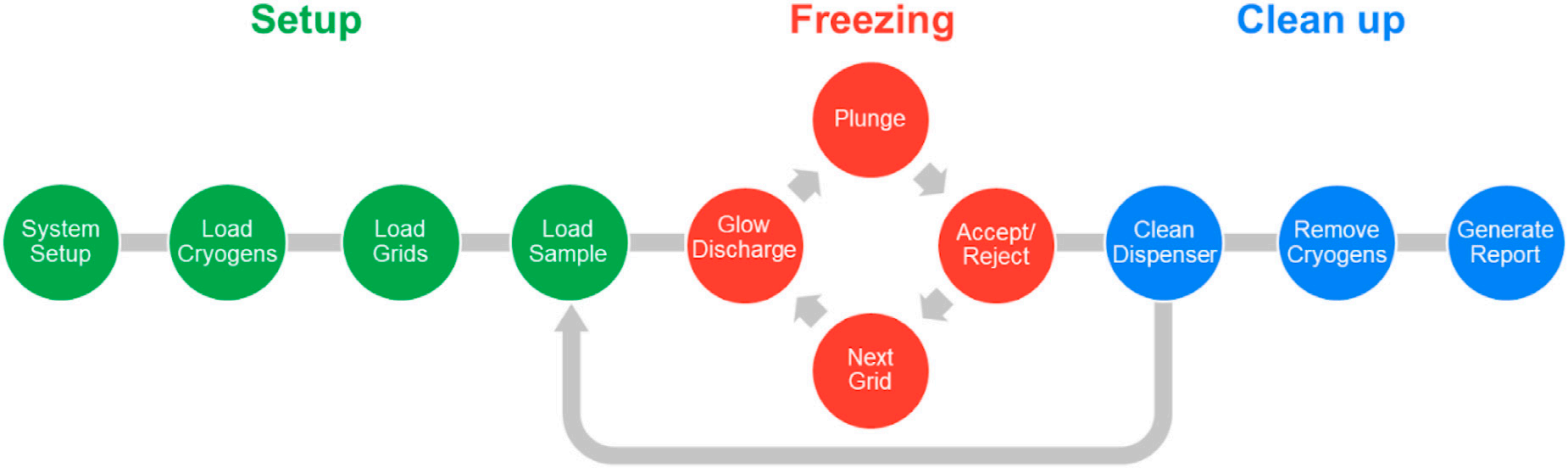
Schematic representation of the chameleon workflow. Steps taken to set up the system are indicated in green. The iterative freezing loop (red) cycles between glow discharging, plunging, accepting/rejecting a grid and then moving on to the next. Each freezing session finishes with clean up steps (blue); however, if a second sample is to be frozen, it can be loaded into the system within the same session, just after the dispenser has been cleaned.

The typical workflow is composed of three main parts: setup, freezing grids, and clean up (Figure 2). During the setup stage, the required reagents (high purity water, a proprietary cleaning solution - chamclean, ethanol, methanol) and consumables (vials, dispenser cleaning pads) are loaded, and the dispenser fluidics system primed in an automated process. The user interface directs the steps necessary to load the cryogens, self-wicking grids, and the sample into the instrument. Up to eight grids can be loaded into the system, of which four can be glow discharged simultaneously. Session and sample information including grid type, sample identity, molecular weight and concentration, and buffer components are recorded to generate a freezing session report. Having set up the system, the specified number of grids are glow discharged, the sample is applied to the grid using the piezoelectric dispenser, and the grid plunge frozen. After freezing, each grid is held in liquid nitrogen and a video of the wicking process can be assessed to decide whether to accept or discard the grid. The system allows full control over glow discharge strength (time and current) and the dispense-to-plunge time which allows optimisation of the specimen preparation process. After grid freezing has been completed the system is cleaned in a semi-automatic manner. The system is then ready for a new sample, or the freezing session can be ended, the accepted grids removed, and a report generated. Generally, a freezing session to produce 6-8 grids of a single sample takes approximately 45 min in its entirety. Switching to a second sample takes approximately 10 min due to the dispenser cleaning protocol, and because many aspects of setting up the system do not need to be repeated, the second sample will require less time to freeze the equivalent number of grids.

**FIGURE 3:**
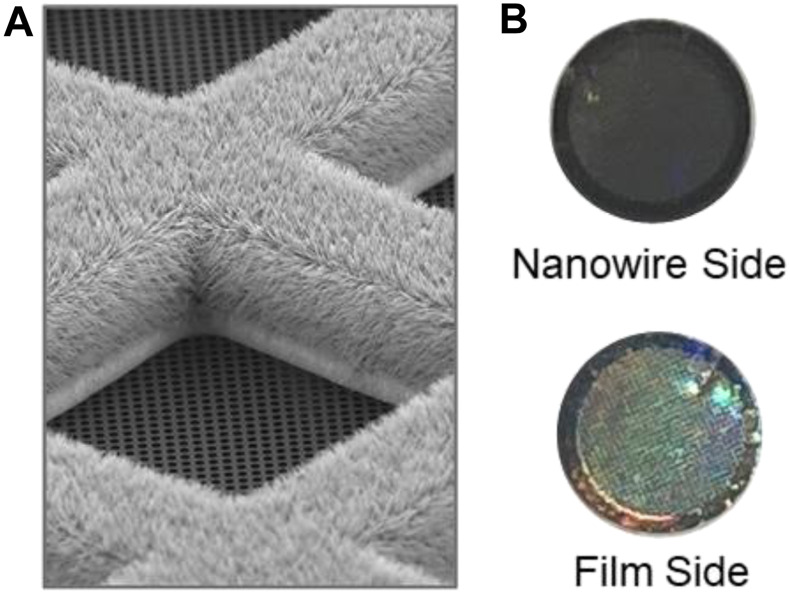
Self-wicking nanowire grids. **(A)**. SEM image showing a grid bar junction with the nanowire side of the grid facing up, with the 1.2 μm diameter holes, 0.8 μm edge-to-edge spacing holey carbon film facing down. **(B)**. The nanowire side of the grid should face towards the dispenser in the chameleon system (to the right). The nanowire side of the grid is characterised by a darker, copper colour, whereas the film side of the grid is characterised by a silver-coloured rim and a more obvious rainbow appearance of the film.

Self-wicking grids and low-volume sample dispensing are key enabling technologies for the chameleon (Jain et al., 2012; Razinkov et al., 2016; Wei et al., 2018). The nanowires provide a controllable wicking mechanism to create a thin film and the low-volume dispenser delivers consistent volumes that do not overwhelm the wicking capacity of the grid. The amount of liquid transported through the nanowires by wicking is a function of the liquid itself and of time, for this reason the time from sample application to frozen grid (dispense-to-plunge time) is a crucial parameter. Self-wicking grids have copper hydroxide nanowires on one side and a holey carbon film on the other (Figure 3A). The holey carbon pattern currently in use is 1.2 μm in diameter with 0.8 μm between each hole (edge-to-edge), producing a more densely packed pattern of holes than is typical. When using these grids, the nanowire side faces towards the dispenser (to the right in the chameleon system). The nanowire side of the grid is generally a darker, copper colour, whereas the film side of the grid is characterised by a silver-coloured rim and a more obvious rainbow appearance of the film (Figure 3B).

## Results and Discussion

### Manufacturing and Quality Control of Self-Wicking Grids

**FIGURE 4:**
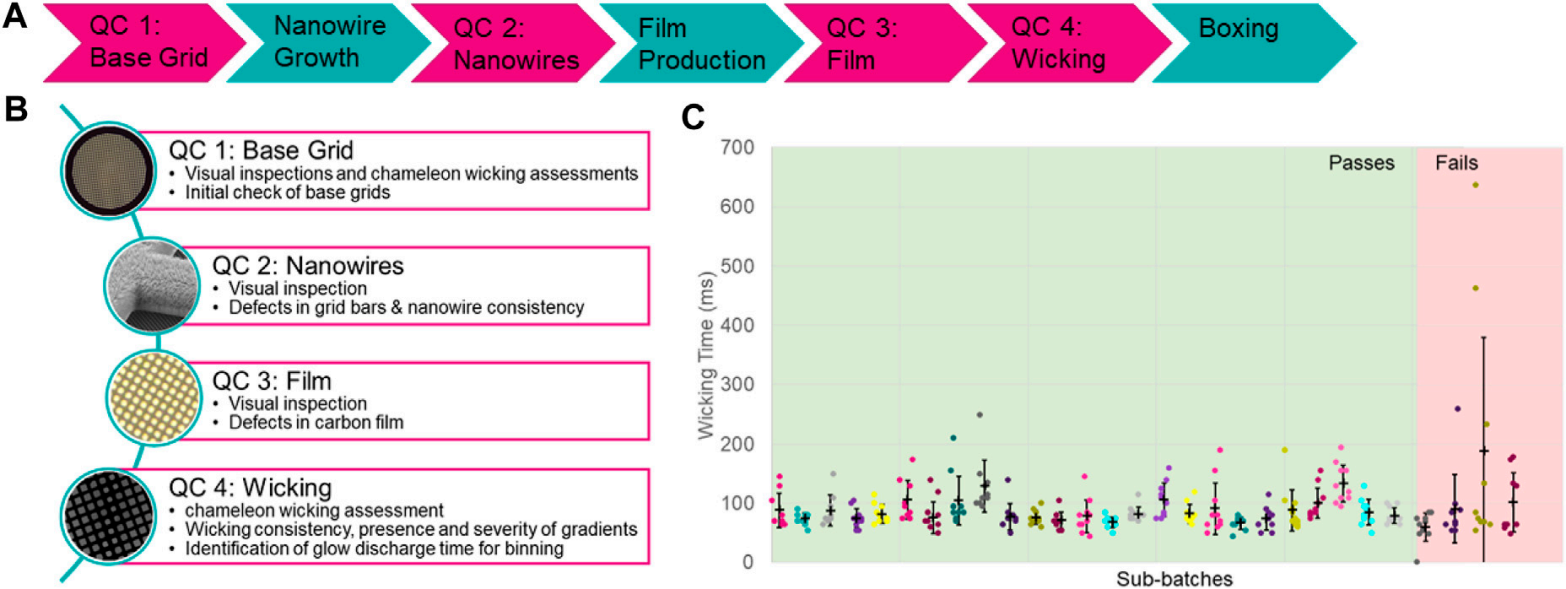
The self-wicking grid manufacturing and quality assessment process. **(A)** A schematic overview of the steps taken to both manufacture (teal) and assess the quality (magenta) of self-wicking grids. **(B)** Descriptions of the quality control checks that are in place. **(C)** Example wicking QC data, indicating sub-batches that have passed or failed. Sub-batches are tested using a standard sample and range of glow discharge times.

The manufacturing process for self-wicking grids requires additional steps when compared to non-self-wicking grids. The challenge is to control the growth of the nanowires and ensure consistency (Figure 4A). Consistency can be assessed through multiple quality control tests integrated into the manufacturing process. The first of these quality checks consists of testing a small subset of starting (base) grids of each batch to identify batches exhibiting the most common manufacturing imperfections (bent or distorted base grids) (Figure 4B). Base grid batches meeting quality criteria enter the full manufacturing process, starting with a series of pre-treatment steps followed by nanowire growth (adapted from Wei et al., 2018). The growth of the nanowires is assessed by a visual inspection using light microscopy of each grid, looking for grid bar defects and nanowire growth consistency. A regular holey plastic film is floated onto the grids, which is then coated in a layer of carbon and the plastic film is dissolved, leaving a thin layer of holey carbon film (adapted from Marr et al., 2014). The carbon film is assessed optically for defects such as tears and pseudoholes in the film of each grid and any such damaged grids are removed.

In the next step of the manufacturing process, ∼10% of final grids from each batch are quality control tested on a chameleon using 5 mg/ml apoferritin in phosphate buffered saline (PBS) to assess glow discharge and wicking behaviour. Following on-board glow discharge that varies between 30–90 s at 12 mA, a single stripe of sample is placed on each grid. The glow discharge settings that result in a wicking time in the range of 60–160 ms are identified. A minimum of 10 grids are then tested at this glow discharge time to assess for consistency and severity of gradients if present (Figure 4C). Subsets of final grids can wick too quickly or too slowly, and these are removed. Only grids in subsets that show a wicking time between 60 and 160 ms when glow discharged for between 30 and 90 s at 12 mA are prepared for use on the instrument.

Employing these grids, the chameleon can provide even a novice user, in less than 2 days, a reliable and straightforward workflow to prepare frozen specimens. We found that a deeper understanding of the interplay between sample characteristics and wicking, and how best to approach a freezing session is beneficial to optimise the process. Here, we describe our current understanding of how these factors interplay and some practical insights into the process.

### Specimen Preparation Using the Chameleon System

**FIGURE 5:**
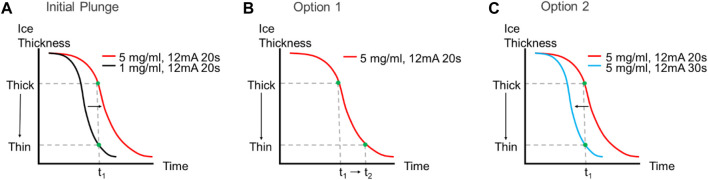
Effect of increased sample concentration on ice thickness and dispense-to-plunge time. **(A)** The same sample at higher concentrations will lead to thicker ice observed on the grid if the dispense-to-plunge time and glow discharge strength are both held constant. There are two options to obtain thinner ice with a more concentrated sample: either increase the dispense-to-plunge time [**(B)**, Option 1] or increase the glow discharge time (or current) and keep the dispense-to-plunge time constant [**(C)**, Option 2].

When a cryoEM project begins with a new sample, different parameters including sample concentration, buffer components, detergent (identity and concentration), and the sample storage/handling temperature need to be optimised. In addition, a range of different blot times or forces, grid types and sample concentrations often need to be evaluated. Multiple iterative rounds of specimen preparation and screening grids are often required to optimise specimen preparation conditions, which is exacerbated by the variability inherent in the standard blot-freezing method (for more information on standard cryoEM methods, see Thompson et al., 2016; Drulyte et al., 2018). The chameleon system does not remove the iterative nature of grid optimisation but makes it faster because the specimen preparation is reproducible with precise control of glow discharge and dispense-to-plunge time (together these influence the ice thickness). We observed that the thickness of ice is inversely proportional to strength of glow discharge (higher current or longer time gives thinner ice) and to the dispense-to-plunge time (longer times give thinner ice) (Figures 5B,C). Thus, in contrast to standard blot-based plungers, the time that grids are glow discharged for and the dispense-to-plunge time are variables that must be empirically determined depending on desired ice thickness and other grid characteristics. Other factors, such as sample viscosity and concentration, influence the precise relationship between glow discharge and dispense-to-plunge time. A more highly concentrated sample will lead to thicker ice for the same glow discharge strength and dispense-to-plunge time (Figure 5A). Empirically exploring these parameters using the chameleon and a screening cryoEM microscope can be required to determine optimal conditions. CryoEM microscope evaluation is essential where particle characteristics such as preferred orientation and/or denaturation at the air-water interface (AWI) (Dandey et al., 2018; Noble et al., 2018b; Klebl et al., 2020; Levitz et al., 2022) are being investigated.

**FIGURE 6:**
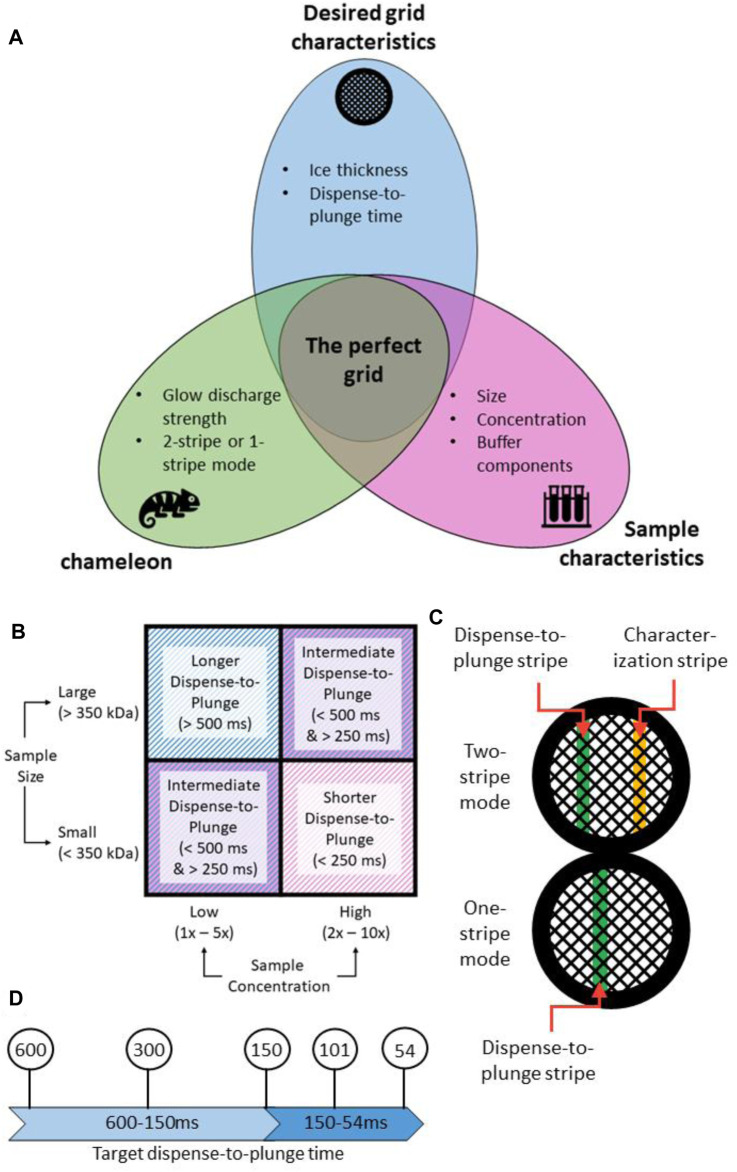
A strategy for approaching chameleon specimen preparation. **(A)** Creation of the “perfect grid” relies on the relationship between the input sample characteristics, the settings used on the chameleon system, and the desired output grid characteristics. **(B)** Sample characteristics such as concentration and size can affect the range of dispense-to-plunge times that are achievable. Shorter dispense-to-plunge times generally require a higher concentration of sample, though this effect is also modulated by the sample size. **(C)** The chameleon system has two modes for grid preparation: one-stripe and two-stripe. In two-stripe mode, a characterization stripe is first applied to determine the ideal wicking time. The second, plunge stripe, is then deposited on a different part of the grid. In one-stripe mode, only one stripe is applied before plunge freezing. **(D)** Any target dispense-to-plunge time can be achieved by adjusting various parameters on the chameleon system. Because of the relationship between dispense-to-plunge time and specimen concentration observed on the grid (see Supplementary Case Study S1), a range of dispense-to-plunge times should be assessed.

The perfect specimen presentation is one that has well separated uniformly sized particles in their native state, embedded in thin ice (<200 nm thick) (Figure 6). Achieving this ideal presentation is dependent on the interplay between the input sample characteristics, the grid preparation device used and the desired output characteristics of the frozen grid (Figure 6). Operational experience of the chameleon system has identified some general trends; the first is that as dispense-to-plunge time decreases, the protein concentration has to be increased for the same number of particles to be seen on the grid (Klebl et al., 2020; Levitz et al., 2022). Additionally, smaller molecular weight samples appear to require less concentration than larger molecular weight samples to achieve the same density of particles on grids (Figure 6B) (Passmore and Russo, 2016).

The chameleon operates over a wide range of buffer conditions, tolerating glycerol and detergents. Especially high sample viscosity may require changes to dispensing parameters such as the amplitude or back pressure used in the piezoelectric dispenser. During the workflow, different sample concentrations or buffer conditions are treated as separate samples with a full cleaning protocol in between to ensure no carryover. Since the sample inside the dispenser equilibrates to ambient temperature, samples that require strict temperature regulation are currently incompatible with this workflow.

**FIGURE 7:**
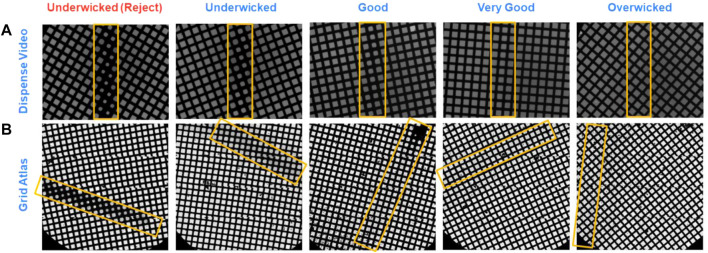
Comparison of grids just prior to freezing on a chameleon and after imaging on a cryo electron microscope. In both two-stripe and one-stripe modes, a video is recorded between sample application and grid freezing that allows the user to judge if the desired film thickness has been obtained. There are five categories a grid can fall into based on its wicking state **(A)**. The **(B)** shows the corresponding grids as imaged on a cryo electron microscope demonstrating the correlation in ice thickness. Stripes in the chameleon dispense video are centrally located and vertical (marked by yellow boxes), whereas the corresponding stripes during imaging (marked by yellow boxes) are randomly located relative to the microscope cryobox and stage.

The current version of the chameleon software (1.12.0) allows for the system to be used in two different modes: two-stripe and one-stripe (Figure 6C). In two-stripe mode, a stripe of sample (the “characterization stripe”) is deposited onto one part of a glow discharged grid. The wicking process is then followed over 2.5 s and a video recorded (Supplementary Video S1). A proprietary algorithm automatically determines the optimal dispense-to-plunge time for the second stripe, including estimation of an offset since wicking occurs more slowly for the second stripe than the first stripe; however, the user can choose a different dispense-to-plunge time. Next, the sample is sprayed onto a different area of the same grid for the second stripe and plunge frozen. The instrument displays another video of the second stripe prior to the grid entering the ethane, allowing the user to assess if the desired film thickness was obtained and to reject grids that do not match the user’s desired film thickness (Figure 7).

In one-stripe mode, only a single stripe of sample is dispensed near the middle of the grid (Figure 6C). The instrument then displays a video of the grid prior to entering the ethane that informs the decision to accept or reject the grid, in the same way as for two-stripe mode (Figure 7). The one-stripe mode provides a 54 ms dispense-to-plunge time option for users attempting to address AWI issues. When using the 54 ms option, the grid does not stop in front of the camera for a video to be collected; instead, a single frame is captured as the grid moves past the camera.

Since the two-stripe mode offers the possibility to view and then vary the dispense-to-plunge time on an individual grid basis, it is often preferred by newer users, but the offset estimate required in this mode can introduce grid-to-grid variability not seen with the one-stripe mode. Because no offset is required in one-stripe mode, shorter dispense-to-plunge times are available (including the 54 ms option); however, prior knowledge about the appropriate glow discharge strength is necessary when using one-stripe mode. These two modes are complementary, and experience suggests a workflow where two-stripe is used initially followed by one-stripe, once the glow discharge strength and dispense-to-plunge time are optimised (see Supplementary Case Study S3).

### Considerations for Specimen Preparation Session

#### Freezing Strategy With no Prior Knowledge

For proteins of molecular weight below 350 kDa, concentrations starting at 1.0 mg/ml and varied down to 0.2 mg/ml empirically encompass most samples tried. For targets above 350 kDa the concentration range of 2.0 mg/ml down to 0.5 mg/ml works well. However, these concentrations can vary for individual samples. Testing with dispense-to-plunge times such as 600, 300, 150, and 101 ms usually provides sufficient insight to optimise the dispense-to-plunge time and concentration necessary (Figure 6D), particularly if the sample displays time-dependent behaviour (migration to the AWI, preferred orientation) on the grids. Supplementary Form S1 is useful for capturing sample information for assessment prior to the freezing session to guide parameter choice.

The optimal glow discharge conditions vary by sample and can shift between grid batches. The default glow discharge conditions of 20 s and 12 mA represent a useful starting point that has been chosen to allow greater control over the wicking process. Both the current and time can be modified as needed to address sample-specific characteristics. For the first grid of a new grid batch or a new sample, it is helpful to use two-stripe mode as the characterization stripe provides additional information on the wicking speed. If the characterization stripe of the first grid wicks more slowly than desired, the glow discharge time can be extended for the second and any subsequent grids. The characterization stripe of two-stripe mode offers the opportunity to view the entire wicking process and evaluate the glow discharge conditions (Supplementary Video S1). Once an appropriate glow discharge strength has been identified, one-stripe mode can be used.

Setting permissive acceptance criteria during the initial investigation allows a wide range of conditions including ice thickness, sample concentrations and dispense-to-plunge times to be evaluated. The specimens can also be screened to determine if a shorter dispense-to-plunge time is imperative to overcome poor sample behaviour such as denaturation/dissociation or preferred orientation; if this is the case and sufficient protein concentrations can be provided, future plunging sessions can focus on achieving grids with shorter dispense-to-plunge times (see *Strategy for a targeted freezing session* below).

#### Freezing Strategy With Prior Knowledge

Where a sample has been screened on a blot-based plunging instrument, this knowledge informs the starting conditions for chameleon sessions. For targets below 350 kDa the concentration of protein needed for the chameleon appears to be approximately equal to the concentration of protein needed for blot-based plunging instruments for longer dispense-to-plunge times (>250 ms). For short dispense-to-plunge times, the concentration should be increased between two and five-fold. Proteins above 350 kDa typically require a two-fold increase in concentration even for dispense-to-plunge times >250 ms compared to blotting. For short dispense-to-plunge times, an increase of up to ten-fold in concentration can be required. For membrane proteins where detergents are present, in some cases a need to double or triple the sample concentration has been observed. However, in individual cases the required concentrations can deviate from these suggestions and further research is required to fully understand these trends.

Where preferred orientation or signs of denaturation at the AWI are known to be a problem, dispense-to-plunge times in the 54–300 ms range are most likely to be successful in overcoming or mitigating these outcomes (see Supplementary Case Studies S1, S2). Improvements in AWI issues have been seen as a gradual shift based on dispense-to-plunge times, with some samples showing improvements only with 54 ms dispense-to-plunge time and others at 150–300 ms dispense-to-plunge times (Klebl et al., 2020; Levitz et al., 2022). Because of the decrease in particles observed on the grid, and the fewer frames available in the dispense-to-plunge video at shorter dispense-to-plunge times, it is recommended to only go as fast as is necessary and to still prepare specimens with a range of dispense-to-plunge times biased to accommodate the available protein concentration. Permissive acceptance criteria are still advisable to provide a range of conditions for the initial screening session.

#### Strategy for a Targeted Freezing Session

Once glow discharge strength, protein concentration, and ice thicknesses have been investigated, the next step is a focused freezing session with stringent grid acceptance criteria and a specific dispense-to-plunge time range (see Supplementary Case Study S3). In general, one-stripe mode is more productive in a targeted freezing session and any grids that do not meet the desired grid characteristics (film thickness, dispense-to-plunge time, enough squares for productive data collection) should be rejected. Where 54 ms dispense-to-plunge time is required, it can be difficult to assess film thickness due to the reduced level of information available in the dispense-to-plunge video. To mitigate this issue, preparing and screening additional grids can help ensure a desired outcome is achieved.

### Best Practices for Imaging Chameleon Grids

**FIGURE 8:**
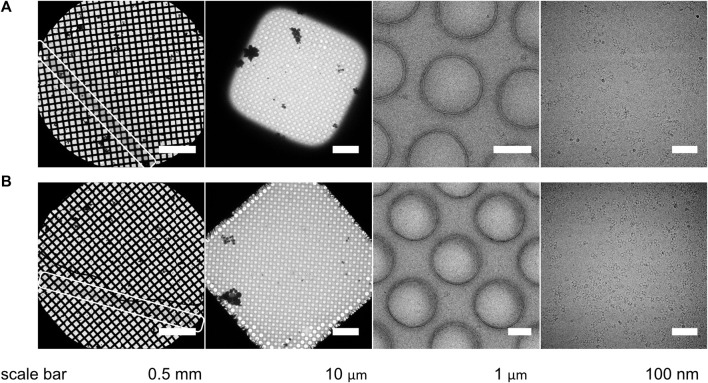
Atlas, grid square, foil hole, and micrograph level images of two different chameleon-plunged grids with the same sample. **(A)** A grid with thicker ice (note the square shown here is not representative of most of the squares within this stripe). **(B)** A grid with thinner ice. The stripes in the atlas-level view are indicated by white rectangles; scale bars are indicated at the bottom.

In either mode of operation, chameleon systems produce a grid with one stripe of vitrified sample that extends the full distance across the grid and is approximately two grid squares wide (Figures 7, 8) (Darrow et al., 2019; Wei et al., 2019). Although there is variability in the measurement due to microscope stage and cryobox configurations, in our hands, this pattern equates to 22 ± 5 grid squares (*n* = 28 grids) in two-stripe mode and 24 ± 5 grid squares (*n* = 54 grids) in one-stripe mode. The remainder of the grid is ‘dry’ which introduces an attraction for contaminating ice crystals. Normally such crystals denote a problematic grid but for chameleon grids, crystals outside the stripe have no practical impact. Usually locating the sample stripe is straightforward at low magnification (135–1×55), however if the grid has been overwicked, higher magnifications (540–940x) may be required (Figure 8). In general, the sample stripe completely covers the grid squares, but partial coverage is also observed, especially with 54 ms dispense-to-plunge time grids. An optimal stripe has consistent ice thickness over the length of the stripe, but thickness gradients (between squares) are also observed. Gradients can be useful during screening as they provide an assessment of the particle distribution relative to ice thickness. Importantly, gradient location and severity can be seen during the screening step on the chameleon, meaning they can be excluded if undesired.

At the grid square level, the ice is thickest in the centre of the grid square and thinnest near the grid bars (Figure 8). Electron imaging of at least two squares per grid (selected from different regions of the stripe) and three holes per square (central area, half-way to the edge, and edge) allows a good assessment of the quality of the grid for structural analysis.

Once a square or squares have been identified to use for data collection, imaging schemes are similar to those used on grids prepared using traditional plunging techniques and collection can be completed as normal (Supplementary Table S1). Occasionally, due to the lack of liquid on the carbon, there is a reverse pattern of intensity such that the holes appear darker than the surrounding carbon necessitating the use of alternative image processing options to reliably find holes. The close spacing of the holes on a chameleon grid allows for more holes to be imaged per stage shift, accelerating data collection (Supplementary Table S1, Supplementary Figure S1). For screening purposes, one grid square will yield 400–700 images. For high-resolution data collection, a chameleon grid imaged at two shots per hole can regularly yield more than 10,000–15,000 images (Figures 7, 8).

The chameleon system is often presented as a next step for users that have encountered difficulties with preferred orientation or AWI denaturation/dissociation using blot-based sample application techniques or as a first step for users anticipating these issues. Although some improvements to complex denaturation or preferred orientation may be noticeable at the micrograph level, the 2D classification step is generally the earliest step that the presence of rare orientations or improvements to complex denaturation can be assessed. Therefore, only by collecting a series of small data sets (∼400–1000 images) at various dispense-to-plunge times can these sample trends be rigorously assessed.

Tomography is still the gold-standard approach for identifying AWI effects as it provides a clear visualisation of the distribution of particles within the ice alongside an empirically determined ice thickness. These pieces of information are crucial in designing future experimental protocols (Noble et al., 2018a; Noble et al., 2018b; Levitz et al., 2022) thus integration of tomographic data collection as a standard part of the single particle data collection process would be ideal.

Even well-behaved samples may still benefit from the chameleon for specimen preparation since it can improve specimen quality in a systematic manner. Quality can be assessed quantitatively by calculating the percentage of particles that contribute to a certain view or final reconstruction, or that are intact versus denatured (Levitz et al., 2022). Higher resolution structures have been determined using many fewer but higher quality specimen particles (Malik et al., 2020).

## Conclusion and the Future of Specimen Preparation

The chameleon system is the first specimen preparation system that provides information about the quality of a grid during the grid preparation process, as opposed to during or after an electron microscopy session. Importantly the chameleon offers the ability to control key experimental variables in a reproducible manner meaning that specimen preparation can begin to move to a more systematic activity where knowledge becomes additive. The systematic nature of the study of specimen preparation will be expanded in the future through additional grid hole sizes and spacings, foil, and support options. As we learn more about the influence of spraying samples, sample and buffer characteristics, dispense-to-plunge times, and AWI interactions on sample quality, the methods for specimen preparation will develop and improve.

The information currently captured during the chameleon specimen preparation process offers future opportunities to increase the throughput of the workflow. We have already shown that sample characteristics (molecular weight, concentration, buffer constituents) help to guide, empirically, the glow discharge parameters and dispense-to-plunge times. Production of robust rules of thumb for operation will require a large increase in the number of samples evaluated. This increase in samples will happen in time as EM continues to dominate experimental structural biology and will afford the opportunity for systematic evaluation of the meta data and chameleon parameters. The increase in samples and data is likely to yield more defined correlations that can be used to make the chameleon system “smarter” and more automated in the future. In addition, the videos and images captured on the chameleon just prior to grid vitrification could inform cryoEM data acquisition, allowing for more automated targeting of appropriate grid squares. This relationship will provide advances in both directions as understanding grid characteristics related to ice thickness, sample concentration and constituents, as well as linked downstream outcomes around particle orientation and denaturation can be fed back into the on-board chameleon image analysis and recommendation algorithms to further reduce the number of screening loops necessary. A key enabling measurement within this relationship is that of ice thickness; due to confounding factors arising from the variability between sample constituents, and the non-routine nature of the analysis, it is not yet a commonly available or reliable measure.

The purpose of robust automation with highly controllable parameters is to shift the effort away from adjusting the biochemical nature of the sample to match a standard freezing protocol and toward using the sample to drive an effective freezing strategy. The systematic exploration of specimen preparation parameters is only possible with the reproducibility of automated systems. Experienced and novice scientists alike will benefit from improved decision-making due to reproduceable outcomes, leading to high-resolution structures more quickly.

## Data Availability

The original contributions presented in the study are included in the article/Supplementary Material, further inquiries can be directed to the corresponding author.
