# Supplementary material for: Approaches to Using the Chameleon: Robust, Automated, Fast-Plunge cryoEM Specimen Preparation
Source: Front Mol Biosci. 2022 Jun 23;9:903148. doi: 10.3389/fmolb.2022.903148 (PMC9260054; doi:10.3389/fmolb.2022.903148)
Supplement: Supplementary file 2 [file DataSheet1.docx]

**Supplementary Information**

**Supplementary Table 1.** Example imaging parameters for a chameleon-plunged grid on either a Talos Arctica (for screening) or a Titan Krios (for high-resolution data collection).

| Imaging Level |  | Talos Arctica G2  (Falcon 3EC camera) | Titan Krios G3i  (K3 camera) |
| --- | --- | --- | --- |
| Atlas | Nominal magnification | 155x | 135x |
|  | Pixel size | 0.562 µm | 0.457 µm |
| Grid Square | Nominal magnification | 940x | 580x |
|  | Pixel size | 31.0 nm | 15.9 nm |
| Foil Hole | Nominal magnification | 11000x | 11500x |
|  | Pixel size | 2.71 nm | 1.62 nm |
| Collection | Nominal magnification | 92000x | 105000x |
|  | Pixel size | 1.5998 Å | 0.869 Å (superresolution) |
| Shots per Hole |  | 1 | 2 |


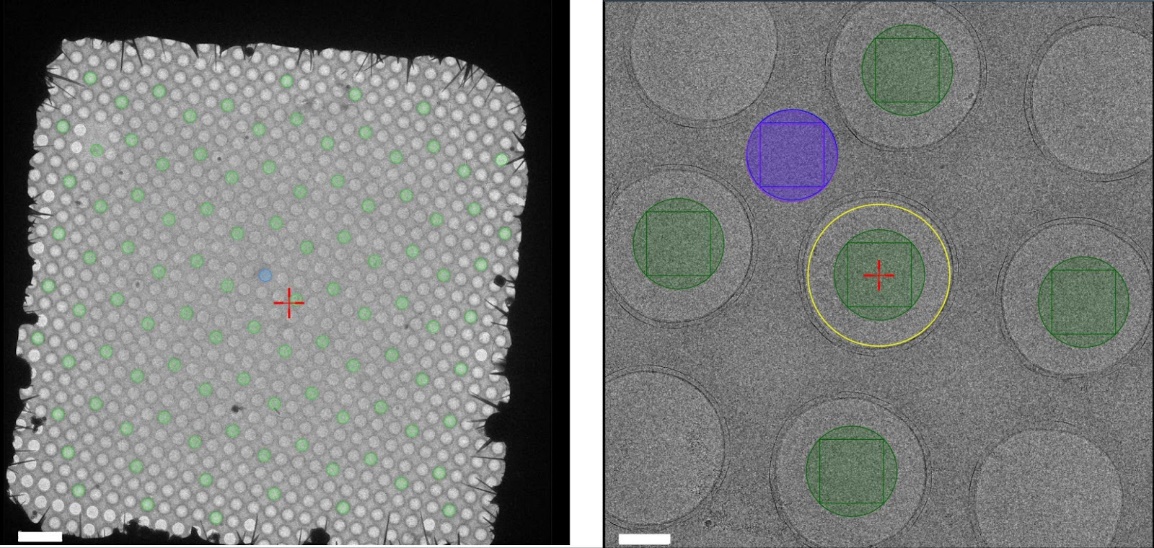


**Supplementary Figure 1.** Representative image of hole selection scheme for screening data set at five shots per stage shift. Green circles represent image acquisition areas per stage shift and the purple circle represents drift / focus area. Scale bar is 5 µm on the left image and 500 nm on the right image. Individual holes are 1.2 µm in diameter with 0.8 µm edge-to-edge spacing in between.

**Case studies**

The following case studies are designed to offer guidance on how to approach issues during a freezing session as well as what to consider between freezing sessions. Each case study, taken from real example data and real freezing sessions, deals with either a common issue encountered during a freezing session or focuses on a particular aim of a freezing session. For each case study, we will refer to Figure 8 in the main text to discuss constraints on and goals of the freezing session.

**Case Study 1: Freezing a concentration-unlimited sample**

If a sample is not concentration-limited, then a freezing session can be performed that varies both *concentration* and *dispense-to-plunge time* for a given sample. A range of ice thicknesses should be accepted as part of the screening process for optimal plunge conditions. The wide range of sample concentrations available as well as the sampling space of the desired grid characteristics means that a wide range of potential combinations of dispense-to-plunge times, ice thicknesses, and sample concentrations can be screened (**Figure 6A, B**). If the sample is suspected to be affected by air/water interface denaturation or preferred orientation, a smaller range of dispense-to-plunge times should be selected (**Figure 6D**). Further screening (to determine correct concentration) and 2D classification (to determine how dispense-to-plunge time affects preferred orientation or air/water interface problems) will determine both the concentration and the dispense-to-plunge time optimal for further targeted freezing sessions.

In the below example, a protein is plunged at concentrations between 1 and 10 mg/mL at varying dispense-to-plunge times, and then subsequently screened for concentration and air/water interface (AWI) effects:

*
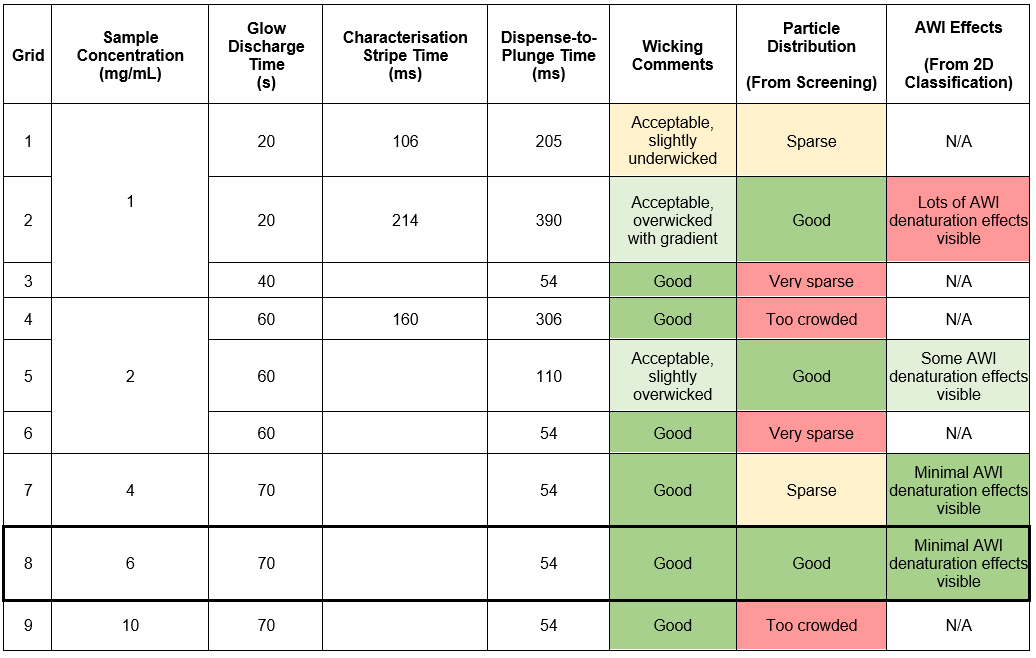
*

In this case, the user should go forward with a targeted freezing session using 6 mg/mL protein and 54 ms dispense-to-plunge time in order to get a data set with good particle concentration on grids as well as minimal air-water interface effects.

Note that although it is true in this case study, a faster dispense-to-plunge time does not always correlate to differences in AWI denaturation or preferred orientation; sometimes there is the opposite or no effect and sometimes the effect is only present at very slow dispense-to-plunge times. Thus, it is imperative that the user empirically determine the effects of dispense-to-plunge time on their sample instead of moving right to the fastest available dispense-to-plunge time.

**Case Study 2: Freezing a concentration-limited sample**

If a sample is concentration-limited, the user should aim for a *wide* *range of dispense-to-plunge times* and accept a *wide range of initial ice thicknesses* to balance the number of particles on the grid and reduce any preferred orientation or air-water interface issues that may be present. The limiting factors of sample concentration and the inherent sample size will mean there is a smaller range of dispense-to-plunge times that will generate useable specimens than in a concentration-limited sample (**Figure 6B**). Screening and/or 2D classification will be necessary to determine the optimal dispense-to-plunge time and ice thickness, at which time a targeted freezing session can be utilized if more data are required (see Case Study 3).

In the below example, a protein is plunged at a single concentration and varying dispense-to-plunge times, and then subsequently screened for concentration and preferred orientation effects:

*
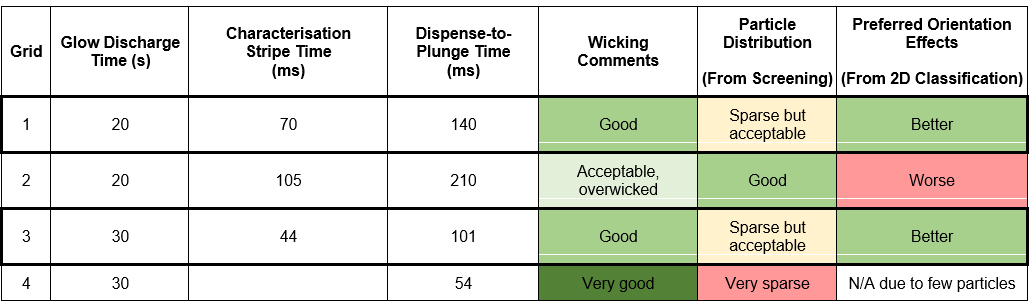
*

Based on the above data, the user should go forward with a targeted freezing session aiming for a dispense-to-plunge time of 101-150 ms.

**Case Study 3: Targeted freezing session**

After initial screenings of dispense-to-plunge times and ice thicknesses for a given sample, the targeted freezing session is aimed at *obtaining multiple grids at an optimal ice thickness and dispense-to-plunge time* (**Figure 6D**). To achieve this, it is best to *use one-stripe mode* as the grids behave more consistently (**Figure 6C**). Unless using the same batch of grids and having already found the glow discharge time that yields the desired wicking time, it is best to start by glow discharging for 20 sec and dispensing a characterisation stripe. Based on the time of the characterisation stripe, more glow discharge can then be added to achieve the desired time in 1-stripe mode. In the following example, a previous screening session (not shown) found that a dispense-to-plunge time around 130 ms resulted in a good particle distribution.


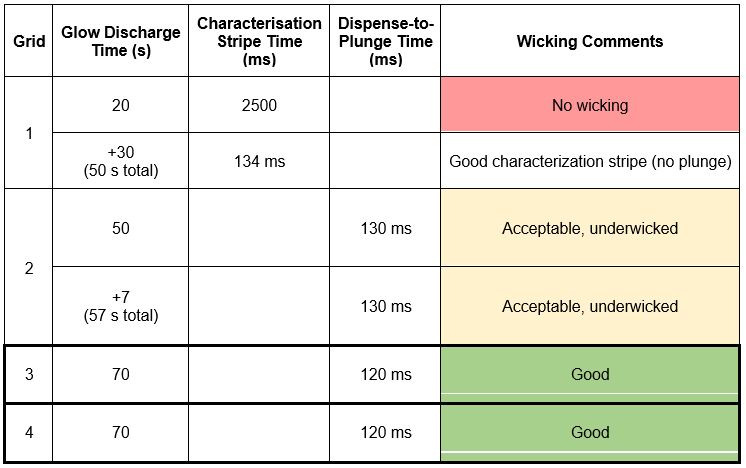


The user now has multiple grids plunged at the correct ice thickness and concentration that can be used for further screening and data collection.

**Supplementary Form 1.** Example sample intake form for chameleon samples.

| Sample Information | |
| --- | --- |
| Sample Name |  |
| Protein Class | Soluble  Membrane  Filamentous |
| Molecular Weight | kDa |
| Sample concentration | mg/ml |
| Buffer Composition | |
| Buffer  (e.g. 20mM Tris) |  |
| pH |  |
| Salt  (e.g. 100mM NaCl) |  |
| Detergent  (e.g. 0.01% (w/v) LMNG) |  |
| Glycerol  (e.g. 0.05% (v/v)) |  |
| Other components  (e.g. 1mM GTP / 2mM MgCl_2_) |  |

| Sample History | |
| --- | --- |
| Sample concentration used previously for cryo grids preparation | mg/ml |
| Freezing instrument(s) used previously  (e.g. Vitrobot, Leica GP) |  |
| Grid Type Used Previously | |
| Base Grid Material | Copper  Gold  Others:____ |
| Mesh Size |  |
| Foil Material | Carbon  Gold  Others:____ |
| Foil Type (Size)  (e.g. holey carbon (1.2/1.3)) |  |
| Support layer  (e.g. continuous carbon/graphene oxide) |  |
| Challenges Experienced in Previous Grid Preparation | |
| Preferential Orientation | Yes  No |
| Air-Water Interface Denaturation | Yes  No |
| Others (please specify) |  |
| Please describe previous attempts to optimize grid preparation  (e.g. negative staining? crosslinking? etc) |  |

**Supplementary Movie 1.** Example of wicking during the characterisation and dispense-to-plunge stripes using 2-stripe mode, including a correlational image of the cryoEM grid atlas.
